# Natural history of Barrett’s esophagus over a 10-year period: a longitudinal endoscopic study in a Japanese population

**DOI:** 10.1007/s00535-026-02495-1

**Published:** 2026-08-04

**Authors:** Kenta Watanabe, Sho Fukuda, Yasutaka Takahashi, So Kodama, Tatsuki Yoshida, Yohei Saruta, Ryo Okubo, Ryo Abe, So Takahashi, Kengo Onochi, Shuichi Ito, Kazuya Kimura, Toshiaki Suzuki, Tamotsu Matsuhashi, Katsunori Iijima

**Affiliations:** 1 Department of Gastroenterology, Akita University Graduate School of Medicine, 1-1-1 Hondo, Akita, 010-8543 Japan; 2 Department of Gastroenterology, Yuri Kumiai General Hospital, Yurihonjo, Akita Japan; 3 Department of Gastroenterology, Omagari Kosei Medical Center, Omagari, Akita Japan; 4 Department of Gastroenterology, Yokote Municipal Hospital, Akita, Japan; 5 Department of Anesthesiology, Honjo-Daiichi Hospital, Akita, Japan; 6 Department of Gastroenterology, Ogaminato Municipal Hospital, Oga, Akita Japan

**Keywords:** Barrett’s esophagus, Endoscopic surveillance, Natural history, Longitudinal study, Japanese population

## Abstract

**Background:**

Since Barrett’s esophagus (BE) is an established precancerous condition for esophageal adenocarcinoma, understanding the natural history of BE is crucial for effective endoscopic surveillance. We aimed to elucidate temporal changes in BE status over a 10-year period using a database derived from repeated endoscopies.

**Methods:**

A total of 9,741 subjects who underwent screening esophagogastroduodenoscopy in 2014 and at least one follow-up examination by December 2025 were included. Transitions in BE status between the index and final examinations were classified as progression, regression, or no change based on differences in BE length. Logistic regression analyses were performed to investigate factors associated with BE progression.

**Results:**

The median follow-up period from the index to final examination was 8.8 years. Overall, BE ≥ 1 cm or BE ≥ 2 cm was newly identified at the final examination in 4.87% and 0.82%, respectively. Progression rates toward longer BE segments remained relatively constant across age groups, ranging from the 30 s to ≥ 70 years. Multivariable regression analyses revealed significant associations between reflux esophagitis and BE progression, with higher risks observed for both Los Angeles grade A and grade B or higher esophagitis.

**Conclusion:**

BE status remained stable in the majority of subjects during approximately nine years of follow-up; however, new BE ≥ 1 cm and ≥ 2 cm developed in 4.87% and 0.82% of subjects, respectively. Such progression occurred irrespective of age at baseline, providing evidence supporting flexible, adaptive surveillance strategies for BE.

**Supplementary Information:**

The online version contains supplementary material available at https://doi.org/10.1007/s00535-026-02495-1.

## Introduction

In Western countries, the incidence of esophageal adenocarcinoma (EAC) has increased markedly since the 1980 s [1, 2]. Consequently, endoscopic surveillance of Barrett’s esophagus (BE)—the only established premalignant condition for EAC [3]—has been implemented to enable early detection of EAC [4]. In general, screening examinations are performed to identify BE in individuals at high risk [5–7], followed by periodic surveillance to facilitate the early diagnosis of neoplastic progression [8]. In Japan, an increase in EAC incidence has been reported since around 2010 [9, 10]. Moreover, the most recent registry-based data through 2024 indicate that the incidence rate has approximately doubled over the past decade [11], highlighting the urgent need to establish appropriate prevention and surveillance strategies tailored to the Japanese population [12].

The risk of EAC is strongly determined by the presence and length of BE, with a longer BE segment conferring a higher risk of malignancy [13–15]. Accordingly, current international guidelines recommend surveillance intervals that vary according to BE length and status [8]. From a practical standpoint, it is therefore crucial to clarify whether BE status remains stable over time or changes with aging. If the BE length remains static after a certain age, a fixed surveillance strategy could be adopted. Conversely, if BE status continues to change dynamically throughout a patient’s lifetime, surveillance strategies must be flexible and responsive to these changes.

Longitudinal studies that repeatedly evaluate the same individuals endoscopically provide direct evidence regarding the natural history of BE development and progression [16–22]. Several such studies have been reported to date, most of which suggest that, once BE develops, its length rarely increases over time [16–18]. However, these studies generally had relatively short follow-up periods—typically no longer than five years—leaving the long-term behavior of BE insufficiently understood [16–22]. In addition, although the prevalence and characteristics of BE are known to vary by ethnicity [12], most previous longitudinal studies were conducted in Western populations, with only limited data available from Japan [19].

We previously analyzed stored esophagogastroduodenoscopy (EGD) images obtained in 2014 from health check-up facilities in Akita Prefecture and reported the prevalence of BE in this population [15, 23]. By systematically reviewing follow-up EGD images from the same individuals, we aimed to elucidate temporal changes in BE status over 10 years, with particular emphasis on the relationship between BE progression and the subjects’ ages at the time of the index EGD.

## Methods

### Study population

In a previous survey, a retrospective analysis was conducted in Akita Prefecture involving 10,166 individuals who underwent screening EGD at five health check-up facilities in 2014 [15, 23]. After applying predefined exclusion criteria, including prior upper gastrointestinal surgery and insufficient follow-up, 9741 subjects with at least one follow-up EGD performed more than one year after the index examination and by December 2025 were included in the present study. The index EGD images obtained and archived in 2014 were previously reviewed to assess the presence and length of BE [15, 23]. In the present study, the most recent EGD images obtained through December 2025 were re-evaluated to examine temporal changes in BE status over approximately 10-year period. This study was approved by the Ethics Committee of Akita University Hospital (approval number AC0014) as well as by the institutional review boards of all participating facilities. Written informed consent was waived owing to the retrospective study design. An opt-out approach was used through public disclosure of study information, allowing participants to decline participation.

### Diagnosis of BE

Following a series of consensus meetings among the raters [15, 23], the index and final follow-up EGD images obtained by December 2025 were evaluated for BE status at each time point. Evaluation in the final EGD images was performed independently, without reference to the index EGD findings. To assess inter-observer reproducibility of BE length classification, four experienced endoscopists independently reviewed a separate validation image set representing a range of BE lengths.

The GEJ was defined as the distal end of the palisade vessels when clearly identifiable. When the palisade vessels could not be identified, the proximal end of the gastric folds, continuous with the gastric lumen, was used as the landmark for the GEJ [24]. BE was diagnosed based on the presence of columnar-lined epithelium extending continuously from the GEJ into the distal esophagus, regardless of length, in accordance with the diagnostic criteria of the Japanese Esophageal Society [25]. The length of the columnar-lined esophagus was assessed using the Prague C and M classification [26]. Participants were categorized according to BE status and maximum circumferential length (M criterion) as follows: absent BE, columnar-lined epithelium < 1 cm (ultra-short-segment Barrett’s esophagus [USSBE]), 1 to < 3 cm (short-segment Barrett’s esophagus [SSBE]), and ≥ 3 cm (long-segment Barrett’s esophagus [LSBE]). For detailed analysis, SSBE was further subdivided into two subcategories: SSBE^−^ (1 to < 2 cm) and SSBE^+^ (2 to < 3 cm).

### Data collection

Clinical data collected at the time of the index health check-up included sex, age, and drinking and smoking habits, body mass index, and waist circumference. Reflux esophagitis (RE) was diagnosed according to the Los Angeles (LA) classification, and the presence of RE was defined as grade A or higher [27]. Endoscopic gastric mucosal atrophy was classified using the Kimura–Takemoto classification system [28].

### Statistical analysis

All statistical analyses were conducted under the assumption that data were missing at random and were handled using multiple imputation with chained Eqs. (30 imputed datasets) [29, 30]. Continuous variables were expressed as medians with interquartile ranges and were compared using Mann–Whitney U test. Categorical variables were summarized as frequencies and proportions and compared using Fisher’s exact test or chi-square test as appropriate. Interobserver agreement was evaluated using weighted kappa statistics, intraclass correlation coefficients, and threshold-based agreement analyses [23, 26]. Changes in BE status between the index and final EGD examinations were classified as progression, regression, or no change, based on differences in BE length. Incidence rates were calculated per 1,000 person-years, and comparisons across follow-up duration categories were performed using Poisson regression. The proportion of BE progression was evaluated across 10-year age groups from 30 s (30–39 years) to ≥ 70 years. Trends in BE progression across these age groups were assessed using Cochran–Armitage trend test. To account for small event numbers, Firth’s penalized likelihood logistic regression was used to investigate factors associated with BE progression. Results were expressed as odds ratios (ORs) with 95% confidence intervals (CIs). All statistical analyses were performed using R software (R Foundation for Statistical Computing, Vienna, Austria) with the EZR graphical interface (Saitama Medical Center, Jichi Medical University, Saitama, Japan) [31]. Multiple imputation and Firth’s penalized likelihood logistic regression were conducted using the mice and logistf packages, respectively. Two-sided *p*-values < 0.05 were considered statistically significant.

## Results

### Baseline characteristics

Of 9,741 subjects enrolled in this analysis, 5,582 (57.3%) were men, with an overall median age of 56 years. The median follow-up period from the index to the final EGD was 8.8 years; 78.1% of subjects had a follow-up duration of at least 5 years, and 26.2% had a follow-up of 10 years or longer. Table 1 summarizes the baseline clinical characteristics according to the five BE categories at the index EGD. The median observation period exceeded 8 years across all BE categories. At the index EGD, absent BE, USSBE, SSBE^−^, SSBE^+^, and LSBE were diagnosed in 5,665 (58.1%), 3,307 (33.9%), 696 (7.1%), 64 (0.66%), and 9 (0.09%) subjects, respectively. At the final EGD, the corresponding prevalences were 6003 (61.6%), 2816 (28.9%), 775 (8.0%), 131 (1.3%), and 16 (0.16%), respectively (Table 1). Interobserver agreement for BE length classification was assessed among the four endoscopists involved in image review (Table 2). The mean weighted kappa value was 0.772 (range, 0.663–0.849), indicating substantial agreement. The intraclass correlation coefficient was 0.782. Agreement for clinically relevant thresholds was 0.618 for < 2 cm versus ≥ 2 cm and 0.769 for < 3 cm versus ≥ 3 cm. Exact agreement was achieved in 55.2% of evaluations, whereas agreement within ± 1 category was achieved in 100%.

**Table 1:** Baseline characteristics of patients according to BE length category at index endoscopy

		BE length category at index endoscopy					*p*-value
		Absent	USSBE	SSBE^-^	SSBE^+^	LSBE	
		*n*=5665	*n*=3307	*n*=696	*n*=64	*n*=9	
Age (years), median (IQR)		54 (46–61)	56 (48–63)	57 (50–64)	54 (47.8–62)	53 (51–62)	<0.001
Age group (years), *n* (%)	<50	1974 (34.8)	981 (29.7)	167 (24.0)	19 (29.7)	2 (22.2)	<0.001
	50–59	2029 (35.8)	1163 (35.2)	258 (37.1)	25 (39.1)	4 (44.4)	
	60–69	1328 (23.4)	896 (27.1)	215 (30.9)	15 (23.4)	3 (33.3)	
	70+	334 (5.9)	267 (8.1)	56 (8.0)	5 (7.8)	0 (0)	
Sex, *n* (%)	Male	2852 (50.3)	2176 (65.8)	495 (71.1)	50 (78.1)	9 (100)	<0.001
	Female	2813 (49.7)	1131 (34.2)	201 (28.9)	14 (21.9)	0 (0)	
Body mass index (kg/m^2^), median (IQR)		22.7 (20.7–25.0)	23.4 (21.4–25.6)	23.3 (21.3–25.5)	22.9 (21.2–24.8)	24.4 (21.1–26.2)	<0.001
Body mass index (kg/m^2^), *n* (%)	<25	4220 (74.5)	2268 (68.6)	487 (70.0)	50 (77.6)	5 (55.6)	<0.001
	≥25	1445 (25.5)	1039 (31.4)	209 (30.0)	14 (22.4)	4 (44.4)	
Waist circumference (cm), median (IQR)		81.5 (75.0–87.6)	83.1 (77.5–89.0)	83.2 (77.7–89.0)	82.9 (75.6–86.7)	87.8 (80.9–93.7)	<0.001
Central obesity^a^, *n* (%)	No	4070 (71.8)	2107 (63.7)	449 (64.6)	43 (66.8)	3 (33.3)	<0.001
	Yes	1595 (28.2)	1200 (36.3)	247 (35.4)	21 (33.2)	6 (66.7)	
Smoking status, *n* (%)	Never	4272 (75.4)	2290 (69.2)	435 (62.4)	43 (67.3)	6 (66.7)	<0.001
	Ever	1393 (24.6)	1017 (30.8)	261 (37.6)	21 (32.7)	3 (33.3)	
Alcohol drinking status, *n* (%)	Never	1580 (27.9)	710 (21.5)	119 (17.1)	9 (14.6)	1 (11.1)	<0.001
	Ever	4085 (72.1)	2597 (78.5)	577 (82.9)	55 (85.4)	8 (88.9)	
RE at index endoscopy, *n* (%)	Absent	5326 (94.0)	2946 (89.1)	580 (83.3)	51 (79.7)	7 (77.8)	<0.001
	LA grade A	260 (4.6)	265 (8.0)	67 (9.6)	6 (9.4)	0 (0)	
	LA grade B+	79 (1.4)	96 (2.9)	49 (7.0)	7 (10.9)	2 (22.2)	
RE at final endoscopy, *n* (%)	Absent	5354 (94.5)	2982 (90.2)	612 (87.9)	51 (79.7)	8 (88.9)	<0.001
	LA grade A	245 (4.3)	263 (8.0)	60 (8.6)	8 (12.5)	1 (11.1)	
	LA grade B+	66 (1.2)	62 (1.9)	24 (3.4)	5 (7.8)	0 (0)	
Gastric atrophy at index endoscopy, *n* (%)	Absent or closed-type	3998 (70.6)	1903 (57.5)	404 (58.0)	44 (68.7)	9 (100)	<0.001
	Open-type	1667 (29.4)	1404 (42.5)	292 (42.0)	20 (31.3)	0 (0)	
Follow-up duration (years), median (IQR)		8.8 (5.4–10.0)	8.8 (5.3–10.0)	9.1 (5.8–10.0)	8.2 (4.7–9.2)	9.8 (8.2–10.2)	<0.001
Follow-up duration category (years), *n* (%)	<5	1238 (21.9)	733 (22.2)	148 (21.3)	18 (28.1)	0 (0)	<0.001
	5–<10	2975 (52.5)	1723 (52.1)	312 (44.8)	37 (57.8)	5 (55.6)	
	≥10	1452 (25.6)	851 (25.7)	236 (33.9)	9 (14.1)	4 (44.4)	
BE length category at final endoscopy, *n* (%)	Absent	4755 (83.9)	1148 (34.7)	100 (14.4)	0 (0)	0 (0)	<0.001
	USSBE	812 (14.3)	1820 (55.0)	184 (26.4)	0 (0)	0 (0)	
	SSBE^-^^b^	92 (1.6)	299 (9.0)	379 (54.5)	4 (6.2)	1 (11.1)	
	SSBE^+^^c^	6 (0.1)	37 (1.1)	32 (4.6)	56 (87.5)	0 (0)	
	LSBE	0 (0)	3 (0.1)	1 (0.1)	4 (6.2)	8 (88.9)	

^a^ Waist circumference of ≥ 85 cm for men and ≥ 90 cm for women

^b^ SSBE with a maximal extent of 1 to < 2 cm

^c^ SSBE with a maximal extent of 2 to < 3 cm

*BE* Barrett’s esophagus; *IQR* interquartile range; *LA* Los Angeles; *LSBE* long-segment Barrett’s esophagus; *RE* reflux esophagitis; *SSBE* short-segment Barrett’s esophagus; *USSBE* ultra-short-segment Barrett’s esophagus

**Table 2:** Interobserver agreement for BE length classification

Agreement measure	Value
Weighted kappa, mean (range)	0.772 (0.663–0.849)
Intraclass correlation coefficient	0.782
Threshold-based agreement	
< 2 cm vs ≥ 2 cm	0.618
< 3 cm vs ≥ 3 cm	0.769
Exact agreement (%)	55.2
Agreement within ± 1 category (%)	100

*BE* Barrett’s esophagus

### Transition of BE status during follow-up

**Fig. 1:**
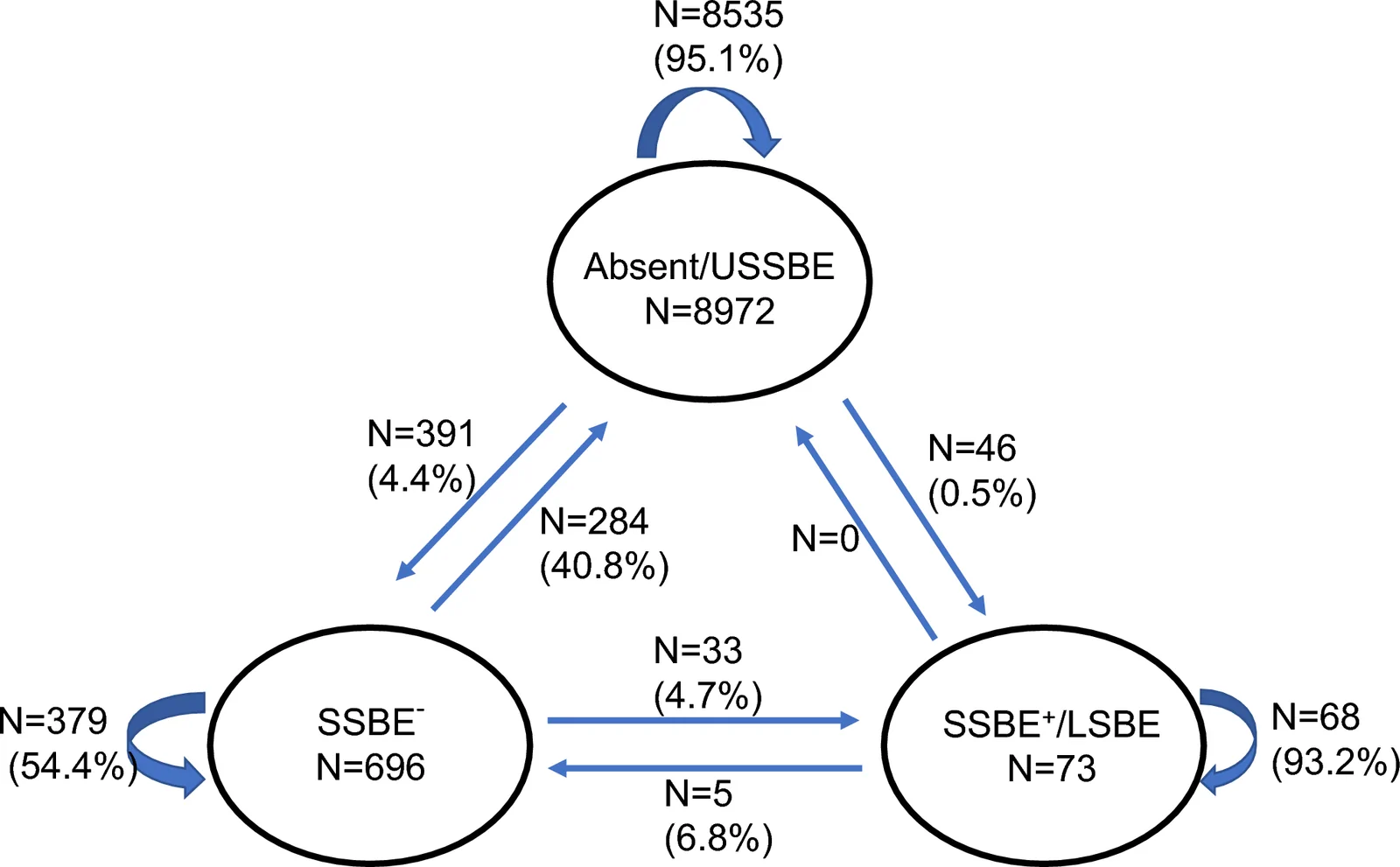
Bidirectional transitions in BE status categories from the index to final examination. BE status was classified into three categories according to segment length: absent/USSBE (0 to < 1 cm), SSBE^−^ (1 to < 2 cm), and "SSBE^+^/LSBE (≥ 2 cm). *BE* Barrett’s esophagus; *USSBE* ultra-short-segment Barrett’s esophagus; *SSBE* short-segment Barrett’s esophagus; *LSBE* long-segment Barrett’s esophagus

Transitions among the five original BE categories during follow-up are shown in Table 1. Because the distinction between absent BE and USSBE showed very low inter-observer agreement [15, 32], these two categories were considered interchangeable. In addition, LSBE was present in only nine subjects at the index examination. Therefore, subsequent analyses were performed using three reclassified BE categories: "absent/USSBE," "SSBE^−^," and "SSBE^+^/LSBE," combining absent BE with USSBE and SSBE^+^ with LSBE. As illustrated in Fig. 1, clear temporal transitions among the 3 BE categories were observed between the index and final EGD. Although absent BE and USSBE were individually interchangeable, the combined "absent/USSBE" category demonstrated high temporal reproducibility, with 95.1% of subjects remaining in this category, whereas 4.4% progressed to SSBE^−^ and 0.5% progressed to "SSBE^+^/LSBE" (BE ≥ 2 cm). Similarly, the "SSBE^+^/LSBE" category showed high temporal stability: 93.2% of subjects remained in the same category, and only 6.8% transitioned to SSBE^−^. In contrast, the SSBE^−^ category showed moderate reproducibility, with 40.8% regressing to "absent/USSBE," 4.7% progressing to "SSBE^+^/LSBE," and 54.4% remaining in the same SSBE^−^ category.

### Age-related progression of BE

**Fig. 2:**
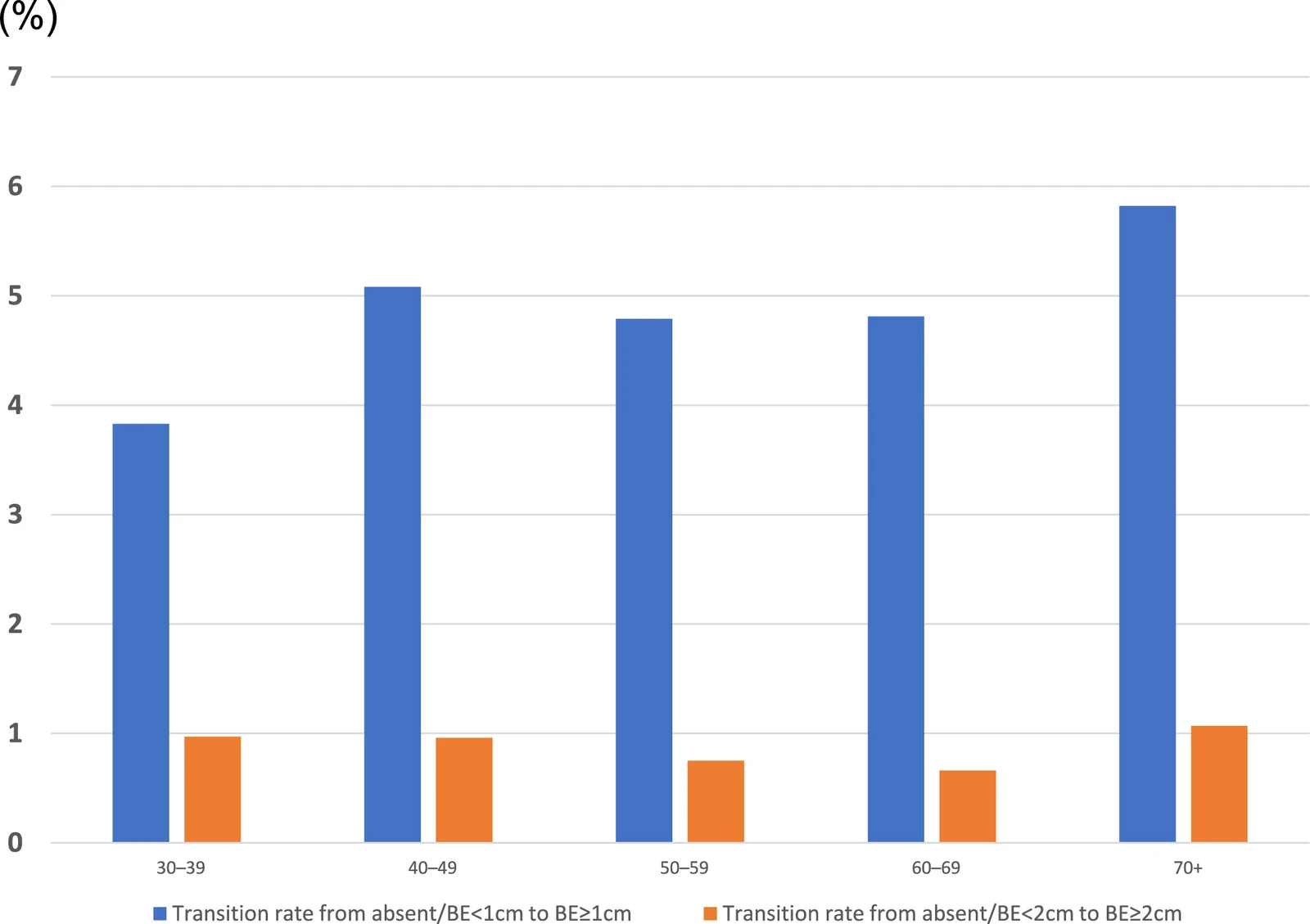
Age-specific proportion of subjects showing progression in BE length. *BE* Barrett’s esophagus

**Fig. 3:**
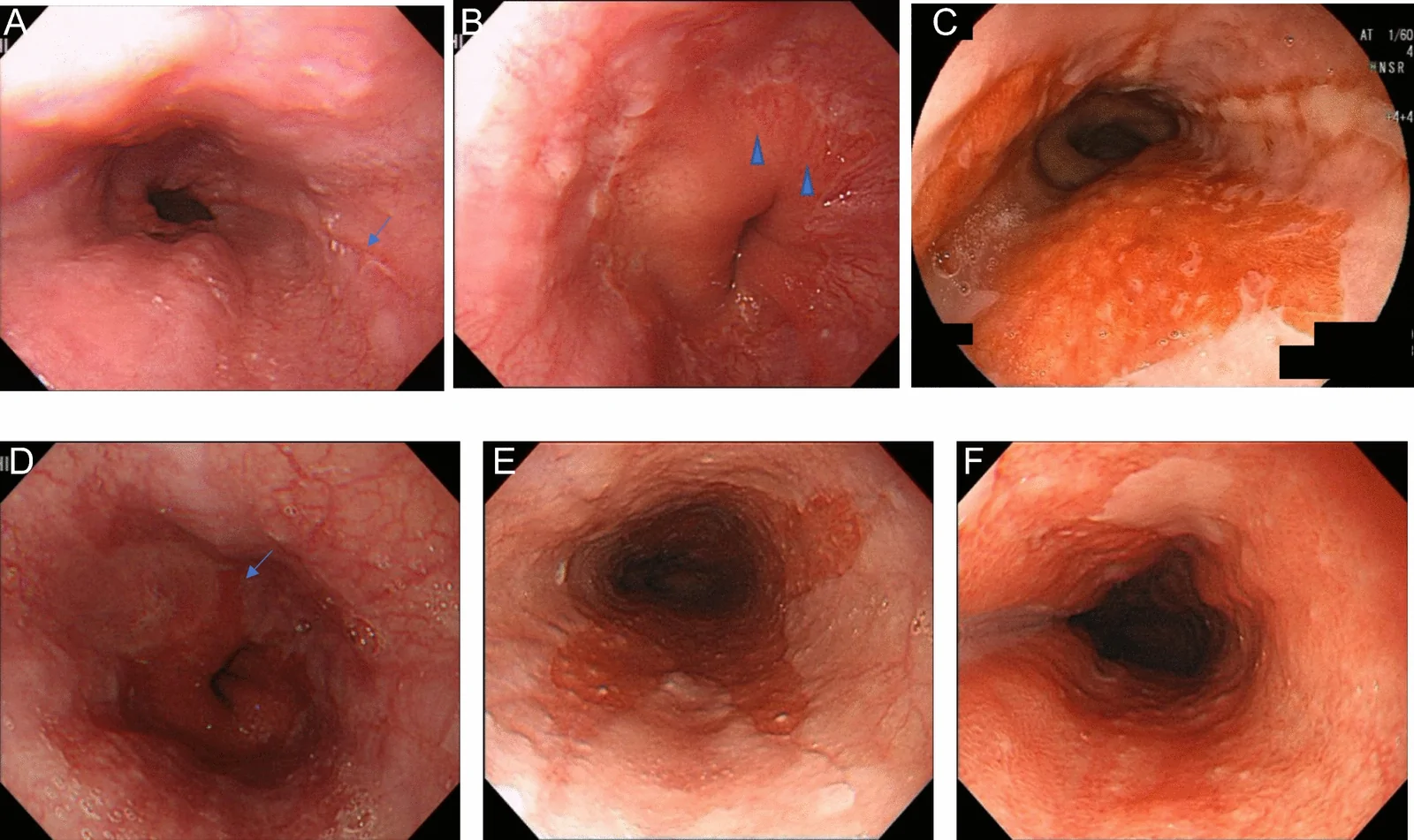
Two representative cases of unequivocal progression of BE during follow-up**.** Case 1: A 72-year-old man (top panels). At the index examination (**A**, **B**), reflux esophagitis (arrow) and USSBE (arrowhead) were observed. At the final examination 73 months later (**C**), a 3-cm LSBE with RE was observed. Case 2**:** A 64-year-old man (bottom panels). A tongue-shaped USSBE (arrow) was observed at the index examination (**D**). At the final examination 111 months later (E, F), an LSBE measuring > 3 cm was observed. *BE* Barrett’s esophagus; *USSBE* ultra-short-segment Barrett’s esophagus; *LSBE* long-segment Barrett’s esophagus; *RE* reflux esophagitis

Overall, BE ≥ 1 cm ("SSBE^−^" plus "SSBE^+^/LSBE") was newly identified at the final EGD in 4.87% of subjects whose baseline status was "absent/USSBE." Conversely, BE ≥ 2 cm ("SSBE^+^/LSBE") was newly developed in 0.82% of subjects whose baseline status was "absent/USSBE" or SSBE^−^. The overall incidence rates of newly developed BE ≥ 1 cm and BE ≥ 2 cm were 6.45 and 1.08 per 1,000 person-years, respectively. When stratified by follow-up duration, the incidence rates of BE ≥ 1 cm were 14.8, 6.29, and 4.68 per 1,000 person-years among subjects with follow-up durations of < 5, 5– < 10, and ≥ 10 years, respectively (*p* < 0.001). Corresponding incidence rates for BE ≥ 2 cm were 1.92, 1.31, and 0.53 per 1,000 person-years, respectively (*p* < 0.001) (Supplementary Table 1). Figure 2 shows the proportion of subjects who newly developed BE ≥ 1 cm or ≥ 2 cm at the final EGD according to 10-year age groups at the index examination. Progression rates toward longer BE remained relatively constant across age groups, ranging from the 30 s to ≥ 70 years. Specifically, the progression rate was approximately 5% for BE ≥ 1 cm and approximately 1% for BE ≥ 2 cm across all age groups. No significant age-related trend was observed for progression to BE ≥ 1 cm or BE ≥ 2 cm (*p* for trend = 0.376 and 0.504, respectively). Representative cases of unequivocal BE progression during follow-up are shown in Fig. 3. During the observation period, Barrett’s adenocarcinoma developed in three subjects. All three patients were male, aged 53, 59, and 70 years at baseline, and their baseline BE status consisted of USSBE (*n* = 1), SSBE^−^ (*n* = 1), and LSBE (*n* = 1).

### Sex-stratified analyses

**Fig. 4:**
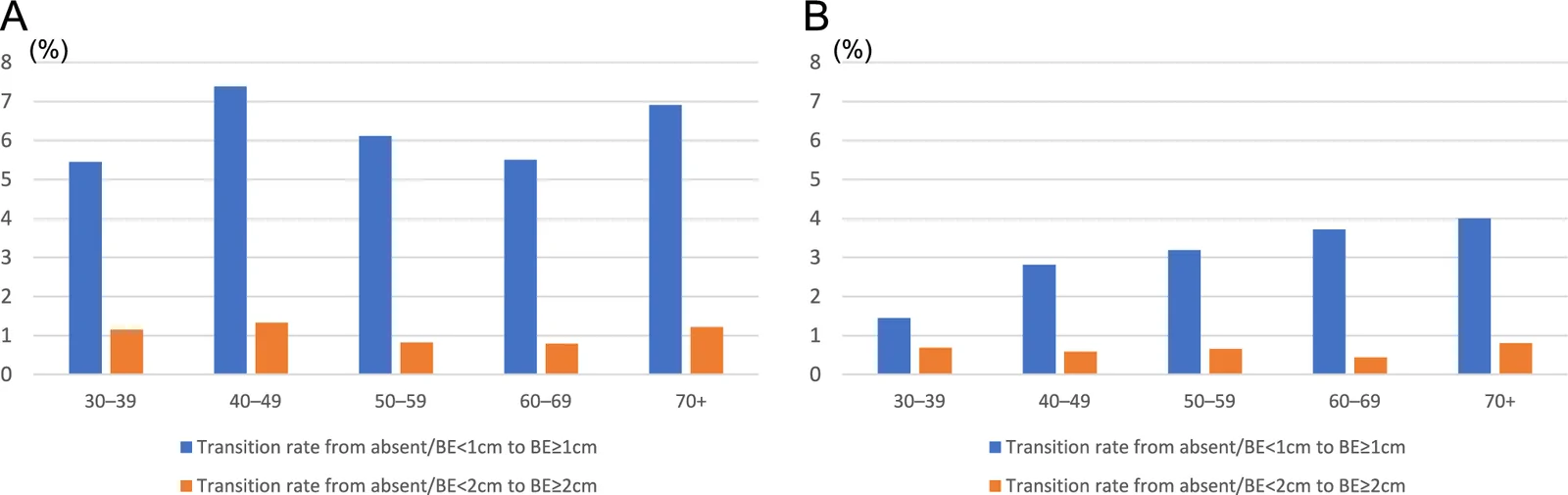
Age-specific proportion of subjects with progression of BE in men (**A**) and women (**B**). *BE* Barrett’s esophagus

Because the prevalence of BE differed markedly between men and women [33], analyses were performed separately by sex (Fig. 4). As shown in Fig. 4, progression to BE ≥ 1 cm or BE ≥ 2 cm was consistently less frequent in women than in men across all age groups. Among men, the incidence of newly developed BE ≥ 1 cm and BE ≥ 2 cm was not associated with age at the index examination (*p* for trend = 0.500 and 0.403, respectively). In contrast, among women, progression to BE ≥ 1 cm occurred more frequently with increasing age at the index examination (*p* for trend = 0.045), whereas progression to BE ≥ 2 cm did not show an age-dependent trend (*p* for trend = 0.865).

### Factors associated with BE progression

Regression analyses were performed to identify clinical factors at the index examination associated with the development of new BE ≥ 1 cm at the final EGD (Table 3). Age at the index examination was not significantly associated with the development of BE ≥ 1 cm in either univariable or multivariable analyses. In the multivariable model, male sex, RE severity, and open-type gastric atrophy at the index EGD were independently associated with progression to BE ≥ 1 cm. Compared with subjects without RE, those with LA grade A and LA grade B or higher had adjusted ORs (95% CIs) of 2.44 (1.79–3.33) and 3.38 (2.17–5.28), respectively. Male sex and open-type gastric atrophy remained independently associated with progression, with adjusted ORs (95% CIs) of 1.64 (1.26–2.13) and 1.34 (1.06–1.69), respectively. In a similar analysis of progression to BE ≥ 2 cm (Supplementary Table 2), both LA grade A and LA grade B or higher were independently associated with progression, with adjusted ORs (95% CIs) of 3.00 (1.60–5.60) and 4.22 (1.84–9.67), respectively. As a supplementary analysis, RE status was further classified according to changes between the index and final examinations (never RE, incident RE, resolved RE, and persistent RE). Compared with subjects who never had RE, incident RE, resolved RE, and persistent RE were all independently associated with progression to BE ≥ 1 cm, with adjusted ORs (95% CIs) of 4.18 (3.12–5.59), 4.44 (2.91–6.78), and 2.77 (2.00–3.83), respectively (Supplementary Table 3).

**Table 3:** Univariable and multivariable logistic regression analyses of factors associated with progression of BE from absent or USSBE to SSBE or LSBE

	Univariable			Multivariable (Firth’s penalized)		
	OR	95% CI	*p*-value	Adjusted OR	95% CI	*p*-value
Age group (years)						
< 50	reference			reference		
50–59	1.00	0.79–1.26	0.982	0.95	0.74–1.21	0.659
60–69	1.00	0.77–1.29	0.992	0.91	0.69–1.20	0.507
70 +	1.22	0.84–1.79	0.296	1.12	0.74–1.70	0.579
Sex						
Female	reference			reference		
Male	2.07	1.67–2.56	< 0.001	1.64	1.26–2.13	< 0.001
RE at index endoscopy						
Absent	reference			reference		
LA grade A	2.53	1.88–3.42	< 0.001	2.44	1.79–3.33	< 0.001
LA grade B +	3.68	2.38–5.70	< 0.001	3.38	2.17–5.28	< 0.001
Gastric mucosal atrophy						
None or closed-type	reference			reference		
Open-type	1.25	1.01–1.53	0.038	1.34	1.06–1.69	0.014
Follow-up duration (years)						
< 5	reference			reference		
5– < 10	1.17	0.91–1.51	0.224	1.18	0.91–1.52	0.204
≥ 10	1.13	0.85–1.51	0.401	1.13	0.84–1.51	0.424
Body mass index (kg/m^2^)						
< 25	reference			reference		
≥ 25	0.98	0.76–1.27	0.898	0.78	0.57–1.07	0.119
Central obesity^a^						
No	reference			reference		
Yes	1.32	1.06–1.65	0.013	1.17	0.87–1.56	0.294
Alcohol drinking status						
Never	reference			reference		
Ever	1.48	1.10–2.00	0.009	1.06	0.77–1.47	0.714
Smoking status						
Never	reference			reference		
Ever	1.49	1.19–1.87	< 0.001	1.20	0.93–1.53	0.155

^a^ Waist circumference of ≥ 85 cm for men and ≥ 90 cm for women

*BE* Barrett’s esophagus; *CI* confidence interval; *LA* Los Angeles; *LSBE* long-segment Barrett’s esophagus; *OR* odds ratio; *RE* reflux esophagitis; *SSBE* short-segment Barrett’s esophagus; *USSBE* ultra-short-segment Barrett’s esophagus

### Factors associated with BE regression

Because regression was frequently observed among subjects with baseline SSBE^−^, an additional analysis was performed to identify factors associated with regression to absent BE or USSBE (Supplementary Table 4). In multivariable analysis, older age was independently associated with regression (60–69 years: adjusted OR 1.73, 95% CI 1.09–2.76; ≥ 70 years: adjusted OR 3.04, 95% CI 1.54–6.00).

## Discussion

This study represents the largest longitudinal investigation to date examining the development and progression of BE using a retrieved endoscopic image database derived from routine health check-up examinations in Japan. By analyzing follow-up EGD images over 10 years, we demonstrated that BE ≥ 1 cm developed in approximately 5% of subjects and new BE ≥ 2 cm in approximately 1% of subjects during follow-up. Notably, BE progression occurred at comparable rates regardless of age at the index examination across a broad age range from the 30 s to ≥ 70 years.

Previous studies from Europe and the United States have generally suggested that BE length reaches its maximum at the time of onset and remains stable thereafter [16–18]. In parallel, epidemiological data indicate that the prevalence of BE plateaus in individuals in their 50 s [34–36], leading to the hypothesis that identifying BE status during a single screening EGD in midlife may be sufficient to determine lifelong EAC risk and surveillance strategies [37]. Under this concept, the absence of BE at screening in one’s 50 s could imply that no further endoscopic surveillance is necessary from the perspective of EAC prevention [35–37]. However, the findings of the present study challenge this assumption. Our data indicate that BE progression does not cease at a specific age but persists at a relatively constant rate over time and, in women, may even increase with advancing age. These observations suggest that BE surveillance strategies should not be fixed based solely on the results of a single examination at a particular age but should instead be adapted flexibly in response to temporal changes in BE status. Interestingly, incidence rates of newly developed BE decreased with longer follow-up duration. One possible explanation is depletion of susceptibles, whereby subjects predisposed to BE development may have developed BE relatively early during follow-up, leaving a population at lower risk over time.

Given the low inter-observer agreement between absent BE and USSBE [15, 32], together with the negligible EAC risk associated with USSBE [15, 23, 38], it is clinically reasonable to consider these two categories as a single group. In the present study, 95% of subjects classified as "absent/USSBE" at baseline remained in the same category over nearly a decade of follow-up, and only 4.4% and 0.5% progressed to BE ≥ 1 cm or BE ≥ 2 cm, respectively. This finding is particularly important because the "absent/USSBE" category accounts for the vast majority of individuals (approximately 80–95%) undergoing screening EGD in Japan [12]. In contrast, subjects with baseline BE ≥ 2 cm ("SSBE^+^/LSBE") exhibited marked temporal stability, with more than 90% remaining in the same category during follow-up and no cases regressing to the "absent/USSBE" category. Because BE ≥ 2 cm has been reported to carry an EAC risk comparable to that of LSBE [39], our findings support the concept that once BE ≥ 2 cm is identified, continued endoscopic surveillance is warranted, despite its low prevalence in screening populations.

In contrast to these relatively stable categories, BE measuring 1 to < 2 cm (SSBE^−^) demonstrated substantial variability over time. Nearly 40% of SSBE^−^ cases regressed to the "absent/USSBE" category, whereas approximately 5% progressed to BE ≥ 2 cm. This variability may be partially attributable to measurement uncertainty, given the narrow length range defining SSBE^−^. From a practical standpoint, the observed instability of SSBE^−^ suggests that rigid surveillance strategies based on a single diagnosis may be inappropriate and that longitudinal reassessment is essential for risk stratification in this subgroup.

The presence and severity of RE at the index examination were strongly associated with subsequent BE progression. Notably, both LA grade A and LA grade B or higher were independently associated with progression, with higher odds ratios observed in subjects with LA grade B or higher esophagitis. This dose–response relationship supports the concept that chronic reflux-induced mucosal injury contributes to the development and elongation of BE. These findings are consistent with previous reports [18–22] and are biologically plausible, as BE is considered an adaptive response to chronic esophageal mucosal injury caused by gastro-esophageal reflux [40]. Clinically, the presence of RE may provide useful additional information when determining surveillance strategies, particularly in patients with SSBE^−^, whose BE status may fluctuate over time.

In addition, open-type gastric atrophy at the index EGD was associated with the development of new BE ≥ 1 cm. This finding appears to contrast with a longitudinal Japanese study reported in 2010, which suggested that the absence of gastric mucosal atrophy was associated with BE progression [19]. One possible explanation for this discrepancy is the temporal shift in *Helicobacter pylori* (*H. pylori*) management in Japan. Since 2013, all *H. pylori*-positive individuals have become eligible for eradication therapy [41], and a substantial proportion of subjects with gastric mucosal atrophy in the present cohort likely underwent eradication during follow-up. Restoration of gastric acid secretion after eradication, particularly in individuals with open-type atrophy, may have contributed to the development of new BE [42, 43]; however, this hypothesis warrants further investigation.

Several longitudinal studies from Western countries have reported no significant elongation of BE over follow-up periods of approximately three years [16–18]. The apparent discrepancy between those findings and the present results may reflect differences in follow-up duration and baseline BE characteristics. Whereas Western studies have predominantly focused on LSBE [16, 17], the current study primarily evaluated BE cases shorter than 3 cm. Taken together, these observations suggest that SSBE has the potential to elongate over time, whereas further elongation may be uncommon once BE exceeds 3 cm.

This study has several limitations. First, the BE diagnosis was based on a retrospective review of archived endoscopic images, which may introduce inter-rater measurement variability. However, similar variability is also inherent in real-world endoscopic measurements, which are typically performed in 1-cm increments [37]. To mitigate this limitation, consensus meetings were held among raters before image evaluation [15, 23]. In addition, inter-observer agreement for BE length classification was substantial (mean weighted kappa, 0.772; intraclass correlation coefficients, 0.782), supporting the reliability of the image-based assessment used in this study. Because the study was based on routine screening endoscopy performed at multiple institutions, minor variations in examination conditions and image acquisition techniques may have existed among endoscopists. Moreover, retrospective image review may be particularly well suited for assessing SSBE, as the entire segment can often be visualized within a single static endoscopic image. Second, because only a small number of subjects had LSBE at baseline, the natural history of cases longer than this is unknown. Third, information regarding *H. pylori* eradication status, use of acid-suppressive therapy (PPI/P-CAB), and hiatal hernia was not uniformly available across participating institutions and therefore could not be included in the analyses. These factors are known to influence GERD and BE development and may have acted as residual confounders. However, in a previous health check-up cohort study conducted in one of the participating institutions, PPI use was observed in only approximately 4–5% of subjects undergoing screening endoscopy [7], suggesting that the impact of acid-suppressive therapy on the present findings may have been limited.

In conclusion, BE status remained stable in the majority of subjects during approximately nine years of follow-up; however, new BE ≥ 1 cm and ≥ 2 cm developed in 5% and 1% of subjects, respectively. Importantly, such progression occurred irrespective of age at baseline. As BE presence and length are the strongest determinants of EAC risk, our findings provide evidence for implementing flexible, longitudinally adaptive surveillance strategies for BE rather than fixed approaches based on a single examination.

## Supplementary Information

Below is the link to the electronic supplementary material.

Supplementary file1 (DOCX 38 KB)

## Abbreviations

BE: Barrette’s esophagus
CI: Confidence interval
EAC: Esophageal adenocarcinoma
EGD: Esophagogastroduodenoscopy
GEJ: Gastro-esophageal junction
LA: Los Angeles
LSBE: Long-segment Barrett’s esophagus
OR: Odds ratio
RE: Reflux esophagitis
SSBE: Short-segment Barrett’s esophagus
USSBE: Ultra-short-segment Barrett’s esophagus
