# Supplementary material for: Natural history of Barrett’s esophagus over a 10-year period: a longitudinal endoscopic study in a Japanese population
Source: J Gastroenterol. 2026 Aug 4;61(10):1408–18. doi: 10.1007/s00535-026-02495-1 (PMC13615931; doi:10.1007/s00535-026-02495-1)
Supplement: Supplementary file 1 [file 535_2026_2495_MOESM1_ESM.docx]

**Supplementary Table 1. Incidence rates of newly developed BE ≥1 cm and BE ≥2 cm according to follow-up duration.**

| **Outcome** | **Follow-up duration (years)** | ***n*** | **Events** | **PY** | **Incidence**  **(per 1000 PY)** | ***p*-value** |
| --- | --- | --- | --- | --- | --- | --- |
| BE ≥1 cm | <5 | 1971 | 86 | 5803 | 14.8 | <0.001 |
|  | 5–<10 | 4698 | 238 | 37819 | 6.29 |  |
|  | ≥10 | 2303 | 113 | 24131 | 4.68 |  |
| BE ≥2 cm | <5 | 2119 | 12 | 6243 | 1.92 | <0.001 |
|  | 5–<10 | 5010 | 53 | 40410 | 1.31 |  |
|  | ≥10 | 2539 | 14 | 26589 | 0.53 |  |

BE, Barrett’s esophagus; PY, person-years.

**Supplementary Table 2. Univariable and multivariable logistic regression analyses of factors associated with progression of BE from absent or USSBE or SSBE^-^ to SSBE^+^ or LSBE.**

|  | **Univariable** | | |  | **Multivariable (Firth's penalized)** | | |
| --- | --- | --- | --- | --- | --- | --- | --- |
|  | **OR** | **95% CI** | ***p*-value** |  | **Adjusted OR** | **95% CI** | ***p*-value** |
| Age group (years) |  |  |  |  |  |  |  |
| <50 | reference |  |  |  | reference |  |  |
| 50–59 | 0.78 | 0.46–1.33 | 0.362 |  | 0.75 | 0.44–1.26 | 0.276 |
| 60–69 | 0.68 | 0.37–1.25 | 0.216 |  | 0.65 | 0.34–1.22 | 0.178 |
| 70+ | 1.11 | 0.49–2.54 | 0.805 |  | 1.09 | 0.46–2.59 | 0.848 |
| Sex |  |  |  |  |  |  |  |
| Female | reference |  |  |  | reference |  |  |
| Male | 1.63 | 1.01–2.62 | 0.045 |  | 1.53 | 0.82–2.87 | 0.185 |
| RE at index endoscopy |  |  |  |  |  |  |  |
| Absent | reference |  |  |  | reference |  |  |
| LA grade A | 2.98 | 1.60–5.57 | <0.001 |  | 3.00 | 1.60–5.60 | <0.001 |
| LA grade B+ | 3.97 | 1.70–9.27 | 0.001 |  | 4.22 | 1.84–9.67 | <0.001 |
| Gastric mucosal atrophy |  |  |  |  |  |  |  |
| None or closed-type | reference |  |  |  | reference |  |  |
| Open-type | 1.01 | 0.63–1.61 | 0.979 |  | 1.22 | 0.73–2.04 | 0.438 |
| Follow-up duration (years) |  |  |  |  |  |  |  |
| <5 | reference |  |  |  | reference |  |  |
| 5–<10 | 1.88 | 1.00–3.52 | 0.050 |  | 1.88 | 1.03–3.44 | 0.040 |
| ≥10 | 0.97 | 0.45–2.11 | 0.946 |  | 0.98 | 0.47–2.07 | 0.962 |
| Body mass index (kg/m^2^) |  |  |  |  |  |  |  |
| <25 | reference |  |  |  | reference |  |  |
| ≥25 | 1.08 | 0.57–2.03 | 0.814 |  | 1.01 | 0.48–2.14 | 0.969 |
| Central obesity^a^ |  |  |  |  |  |  |  |
| No | reference |  |  |  | reference |  |  |
| Yes | 1.14 | 0.60–2.17 | 0.685 |  | 0.89 | 0.40–1.99 | 0.776 |
| Alcohol drinking status |  |  |  |  |  |  |  |
| Never | reference |  |  |  | reference |  |  |
| Ever | 1.18 | 0.57–2.45 | 0.653 |  | 0.91 | 0.42–1.99 | 0.820 |
| Smoking status |  |  |  |  |  |  |  |
| Never | reference |  |  |  | reference |  |  |
| Ever | 1.14 | 0.57–2.29 | 0.702 |  | 0.92 | 0.44–1.92 | 0.833 |

^a^ Waist circumference of ≥85 cm for men and ≥90 cm for women.

BE, Barrett’s esophagus; CI, confidence interval; LA, Los Angeles; LSBE, long-segment Barrett’s esophagus; OR, odds ratio; RE, reflux esophagitis; SSBE, short-segment Barrett’s esophagus; SSBE^-^, SSBE with a maximal extent of 1 to <2 cm; SSBE^+^, SSBE with a maximal extent of 2 to <3 cm; USSBE, ultra-short-segment Barrett’s esophagus.

**Supplementary Table 3. Univariable and multivariable analyses of changes in reflux esophagitis status associated with progression of BE from absent or USSBE to SSBE or LSBE.**

|  | **Univariable** | | |  | **Multivariable (Firth's penalized)** | | |
| --- | --- | --- | --- | --- | --- | --- | --- |
|  | **OR** | **95% CI** | ***p*-value** |  | **Adjusted OR** | **95% CI** | ***p*-value** |
| Age group (years) |  |  |  |  |  |  |  |
| <50 | reference |  |  |  | reference |  |  |
| 50–59 | 1.00 | 0.79–1.26 | 0.982 |  | 0.96 | 0.76–1.23 | 0.761 |
| 60–69 | 1.00 | 0.77–1.29 | 0.992 |  | 0.97 | 0.73–1.29 | 0.848 |
| 70+ | 1.22 | 0.84–1.79 | 0.296 |  | 1.22 | 0.81–1.85 | 0.341 |
| Sex |  |  |  |  |  |  |  |
| Female | reference |  |  |  | reference |  |  |
| Male | 2.07 | 1.67–2.56 | <0.001 |  | 1.52 | 1.16–1.97 | 0.002 |
| RE change |  |  |  |  |  |  |  |
| Never RE | reference |  |  |  | reference |  |  |
| Incident RE | 4.40 | 3.30–5.87 | <0.001 |  | 4.18 | 3.12–5.59 | <0.001 |
| Resolved RE | 4.60 | 3.05–6.95 | <0.001 |  | 4.44 | 2.91–6.78 | <0.001 |
| Persistent RE | 2.80 | 2.05–3.84 | <0.001 |  | 2.77 | 2.00–3.83 | <0.001 |
| Gastric mucosal atrophy |  |  |  |  |  |  |  |
| None or closed-type | reference |  |  |  | reference |  |  |
| Open-type | 1.25 | 1.01–1.53 | 0.038 |  | 1.38 | 1.09–1.75 | 0.007 |
| Follow-up duration (years) | |  |  |  |  |  |  |
| <5 | reference |  |  |  | reference |  |  |
| 5–<10 | 1.17 | 0.91–1.51 | 0.224 |  | 1.14 | 0.88–1.48 | 0.308 |
| ≥10 | 1.13 | 0.85–1.51 | 0.401 |  | 1.13 | 0.84–1.52 | 0.407 |
| Body mass index |  |  |  |  |  |  |  |
| <25 | reference |  |  |  | reference |  |  |
| ≥25 | 0.98 | 0.76–1.27 | 0.898 |  | 0.76 | 0.55–1.05 | 0.096 |
| Central obesity^a^ |  |  |  |  |  |  |  |
| No | reference |  |  |  | reference |  |  |
| Yes | 1.32 | 1.06–1.65 | 0.013 |  | 1.15 | 0.86–1.54 | 0.335 |
| Alcohol drinking status |  |  |  |  |  |  |  |
| Never | reference |  |  |  | reference |  |  |
| Ever | 1.48 | 1.10–2.00 | 0.009 |  | 1.06 | 0.76–1.47 | 0.745 |
| Smoking status |  |  |  |  |  |  |  |
| Never | reference |  |  |  | reference |  |  |
| Ever | 1.49 | 1.19–1.87 | <0.001 |  | 1.19 | 0.93–1.53 | 0.163 |

^a^ Waist circumference of ≥85 cm for men and ≥90 cm for women.

Never RE, no reflux esophagitis at either examination; Incident RE, newly developed reflux esophagitis at the final examination; Resolved RE, reflux esophagitis present at the index examination but absent at the final examination; Persistent RE, reflux esophagitis present at both examinations.

BE, Barrett’s esophagus; CI, confidence interval; LSBE, long-segment Barrett’s esophagus; OR, odds ratio; RE, reflux esophagitis; SSBE, short-segment Barrett’s esophagus; USSBE, ultra-short-segment Barrett’s esophagus.

**Supplementary Table 4. Univariable and multivariable analyses of factors associated with regression of BE from SSBE^-^ to absent BE or USSBE.**

|  | **Univariable** | | |  | **Multivariable (Firth's penalized)** | | |
| --- | --- | --- | --- | --- | --- | --- | --- |
|  | **OR** | **95% CI** | ***p*-value** |  | **Adjusted OR** | **95% CI** | ***p*-value** |
| Age group (years) |  |  |  |  |  |  |  |
| <50 | reference |  |  |  | reference |  |  |
| 50–59 | 1.08 | 0.72–1.63 | 0.713 |  | 1.05 | 0.68–1.61 | 0.822 |
| 60–69 | 1.82 | 1.20–2.77 | 0.005 |  | 1.73 | 1.09–2.76 | 0.021 |
| 70+ | 3.06 | 1.64–5.73 | <0.001 |  | 3.04 | 1.54–6.00 | 0.001 |
| Sex |  |  |  |  |  |  |  |
| Female | reference |  |  |  | reference |  |  |
| Male | 1.26 | 0.90–1.77 | 0.173 |  | 1.04 | 0.69–1.58 | 0.846 |
| RE at index endoscopy |  |  |  |  |  |  |  |
| Absent | reference |  |  |  | reference |  |  |
| LA grade A | 0.77 | 0.45–1.30 | 0.327 |  | 0.92 | 0.53–1.61 | 0.776 |
| LA grade B+ | 0.67 | 0.36–1.24 | 0.202 |  | 0.71 | 0.38–1.35 | 0.301 |
| Gastric mucosal atrophy |  |  |  |  |  |  |  |
| None or closed-type | reference |  |  |  | reference |  |  |
| Open-type | 1.76 | 1.26–2.45 | <0.001 |  | 1.43 | 0.97–2.09 | 0.068 |
| Follow-up duration (years) | |  |  |  |  |  |  |
| <5 | reference |  |  |  | reference |  |  |
| 5–<10 | 0.79 | 0.53–1.17 | 0.240 |  | 0.83 | 0.55–1.25 | 0.378 |
| ≥10 | 1.02 | 0.67–1.54 | 0.939 |  | 1.14 | 0.74–1.76 | 0.542 |
| Body mass index |  |  |  |  |  |  |  |
| <25 | reference |  |  |  | reference |  |  |
| ≥25 | 1.33 | 0.95–1.87 | 0.095 |  | 1.58 | 0.99–2.54 | 0.057 |
| Central obesity^a^ |  |  |  |  |  |  |  |
| No | reference |  |  |  | reference |  |  |
| Yes | 1.20 | 0.87–1.66 | 0.258 |  | 0.87 | 0.55–1.38 | 0.560 |
| Alcohol drinking status |  |  |  |  |  |  |  |
| Never | reference |  |  |  | reference |  |  |
| Ever | 1.14 | 0.71–1.83 | 0.601 |  | 1.10 | 0.65–1.84 | 0.730 |
| Smoking status |  |  |  |  |  |  |  |
| Never | reference |  |  |  | reference |  |  |
| Ever | 1.35 | 0.94–1.95 | 0.100 |  | 1.57 | 1.05–2.36 | 0.029 |

^a^ Waist circumference of ≥85 cm for men and ≥90 cm for women.

BE, Barrett’s esophagus; CI, confidence interval; LA, Los Angeles; LSBE, long-segment Barrett’s esophagus; OR, odds ratio; RE, reflux esophagitis; SSBE, short-segment Barrett’s esophagus; SSBE^-^, SSBE with a maximal extent of 1 to <2 cm; USSBE, ultra-short-segment Barrett’s esophagus.
